# Association between the triglyceride–glucose index and hypertension: potential role of fatty liver in a cross-sectional study

**DOI:** 10.3389/fendo.2026.1826006

**Published:** 2026-05-08

**Authors:** Yuzhan Lin, Ruixue Sun, Lifeng Wu, Lei Chen, Chen Fu, Shaorong Yan, Jianze Cai

**Affiliations:** 1 Department of Clinical Laboratory, The Third Affiliated Hospital of Wenzhou Medical University, Ruian, Zhejiang, China; 2 Department of Clinical Laboratory, Ruian Traditional Chinese Medicine Hospital, Ruian, Zhejiang, China; 3 Hospital Infection Management Department (Public Health Department), The Third Affiliated Hospital of Wenzhou Medical University, Ruian, Zhejiang, China

**Keywords:** cross-sectional study, fatty liver, hypertension, insulin resistance, triglyceride–glucose index

## Abstract

**Background:**

The triglyceride–glucose (TyG) index is widely recognized as a surrogate marker of insulin resistance and reflects disturbances in glucose and lipid metabolism. Although higher TyG index levels have been associated with hypertension, the factors related to this association remain incompletely understood. This study aimed to examine the association between the TyG index and hypertension and to explore whether fatty liver might statistically account for part of this association.

**Methods:**

This cross-sectional study included participants who underwent routine health examinations at the Third Affiliated Hospital of Wenzhou Medical University between 2021 and 2025. For individuals with multiple examination records during the study period, only the first record was retained for analysis. The TyG index was calculated using fasting triglyceride and glucose levels. Fatty liver was assessed by abdominal ultrasonography, and hypertension was defined according to the 2023 European Society of Hypertension guidelines. Multivariable logistic regression models were used to evaluate the associations of the TyG index with hypertension and fatty liver. Mediation analysis was performed to explore whether fatty liver might account for part of the observed association between the TyG index and hypertension.

**Results:**

A total of 115,327 participants were included in the final analysis. The TyG index was significantly associated with hypertension. Each 1-unit increase in the TyG index was associated with higher odds of hypertension (adjusted OR = 2.09, 95% CI: 2.03–2.15). The TyG index was also strongly associated with fatty liver (adjusted OR = 7.05, 95% CI: 6.82–7.28). Mediation analysis suggested that fatty liver might account for part of the observed association between the TyG index and hypertension, with a proportion statistically accounted for of 23.50% (95% CI: 21.39%–25.96%).

**Conclusions:**

In this health examination population, a higher TyG index was significantly associated with hypertension, and fatty liver may account for part of the observed association. Further longitudinal studies are needed to clarify these findings.

## Introduction

Hypertension remains a major global public health concern, with persistent disparities in prevalence, treatment, and control across populations. In the United States, undiagnosed or uncontrolled hypertension contributes to heightened cardiovascular disease risk and substantial healthcare costs, underscoring the urgent need for integrated clinical and community-based management strategies (1). Population-based studies in the Brazilian Amazon have identified hypertension as one of the most prevalent chronic conditions, frequently coexisting with other non-communicable diseases and linked to socioeconomic determinants of health (2). Furthermore, individuals with hypertension have demonstrated significantly increased utilization of urgent, emergency, and specialized healthcare services following COVID-19 infection, reflecting the ongoing disease burden and complex healthcare needs of this population (3).

The triglyceride-glucose (TyG) index, calculated from fasting triglyceride and glucose levels, is widely regarded as a simple and cost-effective surrogate marker of insulin resistance (IR) and has also been considered an indicator of glucose and lipid metabolic dysregulation (4). Accumulating evidence suggests that IR and disordered glucose-lipid metabolism are closely related to elevated cardiovascular risk, with the TyG index serving as a practical marker of these metabolic abnormalities (5). In addition to cardiovascular manifestations, metabolic dysfunction is closely associated with hepatic abnormalities such as fatty liver, which is increasingly recognized as an important component of systemic metabolic disorders. Previous studies have shown that IR and abnormal lipid metabolism are closely associated with fatty liver and its related cardiometabolic complications (6). Moreover, the TyG index has been associated with arterial stiffness, an important feature related to elevated blood pressure and vascular dysfunction (7). Given its accessibility and strong associations with multiple metabolic and cardiovascular pathways, the TyG index holds substantial clinical value for identifying individuals at elevated risk of metabolic and cardiovascular disorders in routine practice.

Emerging epidemiological evidence has consistently demonstrated an association between higher TyG index levels and hypertension. A prospective cohort study based on the China Health and Nutrition Survey reported that higher TyG index levels were associated with an increased risk of incident hypertension over 6 years of follow-up (8). Another long-term longitudinal study further suggested that the cumulative burden of body mass index was associated with hypertension risk partly through the TyG index (9). In addition, accumulating evidence suggests that the TyG index is associated with early manifestations of vascular injury, including carotid atherosclerosis and plaque formation. Our previous studies also identified sex-specific associations between the TyG index, carotid plaque, and new-onset hypertension in Chinese populations (10–12).

Despite these findings, the factors related to the association between the TyG index and hypertension remain incompletely understood. Prior studies have mainly focused on insulin resistance-related mechanisms (13), whereas the possible contribution of hepatic metabolic abnormalities, particularly fatty liver, has received relatively limited attention. Because the TyG index is widely regarded as a surrogate marker of insulin resistance and glucose–lipid metabolic dysregulation, exploring whether fatty liver may be involved in the association between the TyG index and hypertension may help to further clarify the metabolic relationship among dysregulated glucose–lipid metabolism, fatty liver, and hypertension. Therefore, the present study aimed to investigate the association between the TyG index and hypertension and to examine whether fatty liver may partly account for this observed association in mediation analysis.

## Methods

### Ethics statement

This study was approved by the Institutional Review Board of the Third Affiliated Hospital of Wenzhou Medical University. Informed consent was waived by the review board due to the retrospective nature of the study and the use of fully anonymized data, which posed no identifiable risk to participants (YJ2026011). All study procedures were conducted in accordance with the ethical principles outlined in the Declaration of Helsinki.

### Study design and data source

This cross-sectional study utilized data from annual physical examination participants at the Third Affiliated Hospital of Wenzhou Medical University between January 1, 2021, and December 31, 2025. The dataset included demographic information, physical examination findings, laboratory measurements, and questionnaire-based lifestyle data collected as part of routine clinical practice.

### Study population

**Figure 1:**
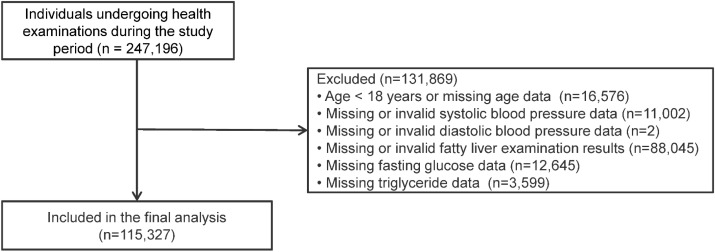
Flowchart of participant selection. For individuals with multiple examination records during the study period, only the first record was retained for analysis.

The study population initially consisted of 247,196 individuals who underwent routine health examinations at the Third Affiliated Hospital of Wenzhou Medical University during the study period. For individuals with multiple examination records, only the first record was retained for analysis. Individuals were excluded if they met any of the following criteria (1): age < 18 years or missing age data (2); missing or invalid systolic blood pressure data (3); missing or invalid diastolic blood pressure data (4); missing or invalid fatty liver examination results (5); missing fasting glucose data; or (6) missing triglyceride data. After these exclusions, 115,327 participants were included in the final analysis. The participant selection process is shown in **Figure 1**.

### Exposure assessment

The primary exposure was the triglyceride-glucose index, calculated using the validated formula: ln[triglyceride (mg/dL) × fasting plasma glucose (mg/dL)/2] (14). Venous blood samples were collected from participants after an overnight fast of at least 8 hours, and triglyceride and fasting plasma glucose levels were measured using standard automated laboratory assays.

### Outcome definition

The primary outcome was hypertension, defined according to the 2023 European Society of Hypertension guidelines (15) as a systolic blood pressure ≥140 mmHg, a diastolic blood pressure ≥90 mmHg, self-reported physician-diagnosed hypertension, or current use of antihypertensive medications. Blood pressure was measured using a calibrated sphygmomanometer with the participant in a seated position after at least 5 minutes of rest, following standardized clinical procedures.

### Assessment of fatty liver

Fatty liver was assessed using abdominal ultrasonography performed during routine health examinations by experienced radiologists. The diagnosis of fatty liver was based on standard ultrasonographic criteria, including increased hepatic echogenicity compared with the renal cortex, attenuation of the ultrasound beam, and poor visualization of intrahepatic structures (16). Participants who met these imaging criteria were classified as having fatty liver.

### Covariate assessment

Potential confounding covariates were collected, including demographic factors and lifestyle behaviors. Demographic covariates included age, sex, marital status, and ethnicity. Lifestyle factors included physical activity level, smoking status (categorized as non-smoker, former smoker, or current smoker), and alcohol consumption status (categorized as non-drinker, former drinker, or current drinker), all assessed via self-reported questionnaires during the physical examination. Family history of hypertension and self-reported dietary preference were additionally considered in sensitivity analyses.

### Data processing

Demographic information, physical examination findings, and laboratory test results were merged to construct an integrated analytical dataset. Participant exclusions were applied as described in the Study Population section. Missing data in physical activity, smoking status, and alcohol consumption were handled using the missing-indicator method.

### Statistical analysis

Continuous variables were expressed as mean ± standard deviation (SD), and categorical variables were presented as numbers (percentages). Differences between participants with and without hypertension were evaluated using the t-test or non-parametric tests (Wilcoxon rank-sum test) for continuous variables, and the chi-square test or Fisher’s exact test for categorical variables, as appropriate.

Multivariable logistic regression analyses were performed to examine the associations of the TyG index with hypertension and fatty liver, with odds ratios (ORs) and 95% confidence intervals (CIs) reported. Multiple models were constructed with progressive adjustment for potential confounders: the non-adjusted model included no covariates; Model 1 was adjusted for age and sex; and Model 2 was further adjusted for marital status, ethnicity, physical activity, alcohol consumption, and smoking status.

In addition, mediation analysis was conducted to explore whether fatty liver might statistically account for part of the association between the TyG index and hypertension (17). Because hypertension was a binary outcome, the mediation analysis used generalized linear models with a probit link for the outcome model, and the total, direct, and indirect effects were estimated as differences in the predicted probability of hypertension under the treatment contrast specified in the mediation analysis. We reported the average total, direct, and indirect effects, together with the proportion statistically accounted for, with 95% confidence intervals and P values estimated by nonparametric bootstrap with 1,000 resamples. Given the cross-sectional design, this mediation analysis was intended as a statistical exploration of the observed association rather than evidence of a causal mediating effect.

A directed acyclic graph was constructed to clarify the hypothesized relationships among the TyG index, fatty liver, hypertension, and covariates, and is presented in **Supplementary Figure 1**.

A two-sided P value < 0.05 was considered statistically significant. All statistical analyses were performed using R version 4.5.0.

## Results

### Study population and baseline characteristics

**Figure 1** presents the participant selection process. A total of 247,196 individuals who underwent routine health examinations between 2021 and 2025 were initially screened. For individuals with multiple examination records during the study period, only the first record was retained for analysis. After applying the exclusion criteria, 115,327 participants were included in the final analysis. Baseline characteristics of the study population stratified by hypertension status are summarized in **Table 1**. Compared with participants without hypertension, those with hypertension were older (56.79 ± 13.64 vs 43.63 ± 13.43 years), more likely to be male (58.31% vs 46.38%), and had a higher prevalence of fatty liver (52.88% vs 30.98%) and a higher TyG index (8.87 ± 0.62 vs 8.51 ± 0.60) (all P < 0.001). Participants with hypertension also exhibited higher levels of fasting glucose, triglycerides, total cholesterol, low-density lipoprotein cholesterol, uric acid, and liver enzymes (all P < 0.001). In addition, current alcohol drinking was more common in the hypertension group, whereas physical activity and smoking status did not differ significantly between the two groups.

**Table 1:** Baseline characteristics of participants according to hypertension status.

Variable	Non-hypertension (n = 87,137)	Hypertension (n = 28,190)	Standardized difference	P value
Age, years	43.63 ± 13.43	56.79 ± 13.64	0.97	<0.001
Height, cm	161.33 ± 8.07	159.97 ± 8.17	0.17	<0.001
Weight, kg	61.37 ± 10.20	62.24 ± 10.44	0.09	<0.001
Body mass index, kg/m²	23.51 ± 2.97	24.24 ± 3.04	0.24	<0.001
Waist circumference, cm	79.65 ± 8.47	81.40 ± 8.51	0.21	<0.001
Hip circumference, cm	91.52 ± 6.55	91.71 ± 6.75	0.03	0.073
Systolic blood pressure, mmHg	117.21 ± 12.35	152.52 ± 12.98	2.79	<0.001
Diastolic blood pressure, mmHg	70.27 ± 8.86	86.89 ± 10.65	1.70	<0.001
Male, n (%)	40,411 (46.38%)	16,438 (58.31%)	0.24	<0.001
Married, n (%)	75,794 (87.15%)	27,148 (96.53%)	0.35	<0.001
Han ethnicity, n (%)	74,003 (98.77%)	24,767 (99.10%)	0.03	<0.001
Fatty liver, n (%)	26,991 (30.98%)	14,908 (52.88%)	0.46	<0.001
Physical activity, n (%)			0.05	0.683
Inactive	687 (57.25%)	221 (54.98%)		
Occasional	466 (38.83%)	166 (41.29%)		
Regular	47 (3.92%)	15 (3.73%)		
Alcohol drinking, n (%)			0.17	0.005
No	1,095 (87.74%)	337 (81.60%)		
Former	4 (0.32%)	2 (0.48%)		
Current	149 (11.94%)	74 (17.92%)		
Smoking status, n (%)			0.01	0.965
No	1,147 (91.83%)	381 (91.59%)		
Former	3 (0.24%)	1 (0.24%)		
Current	99 (7.93%)	34 (8.17%)		
TyG index	8.51 ± 0.60	8.87 ± 0.62	0.59	<0.001
Fasting glucose, mmol/L	4.86 ± 1.01	5.48 ± 1.50	0.49	<0.001
Triglycerides, mmol/L	1.55 ± 1.33	1.99 ± 1.64	0.29	<0.001
Total cholesterol, mmol/L	4.90 ± 0.95	5.15 ± 1.04	0.25	<0.001
LDL-cholesterol, mmol/L	3.08 ± 0.89	3.26 ± 0.99	0.19	<0.001
HDL-cholesterol, mmol/L	1.29 ± 0.33	1.23 ± 0.32	0.17	<0.001
Uric acid, μmol/L	341.55 ± 93.16	364.56 ± 96.42	0.24	<0.001
Creatinine, μmol/L	63.67 ± 16.52	67.75 ± 22.12	0.21	<0.001
ALT, U/L	27.21 ± 28.74	32.51 ± 29.89	0.18	<0.001
AST, U/L	24.50 ± 17.04	28.21 ± 17.19	0.22	<0.001
GGT, U/L	32.03 ± 41.54	46.38 ± 64.53	0.26	<0.001

Values are presented as mean ± standard deviation (SD) for continuous variables and number (percentage) for categorical variables. P values were calculated using the t-test or Wilcoxon rank-sum test for continuous variables and the χ² test or Fisher’s exact test for categorical variables as appropriate. Physical activity, alcohol drinking, and smoking status had substantial missing data. Percentages for these variables were calculated among participants with available data.TyG, triglyceride–glucose index; BMI, body mass index; SBP, systolic blood pressure; DBP, diastolic blood pressure; ALT, alanine aminotransferase; AST, aspartate aminotransferase; GGT, gamma-glutamyl transferase; LDL-C, low-density lipoprotein cholesterol; HDL-C, high-density lipoprotein cholesterol.

### Distribution of hypertension and fatty liver across TyG quartiles

**Figure 2:**
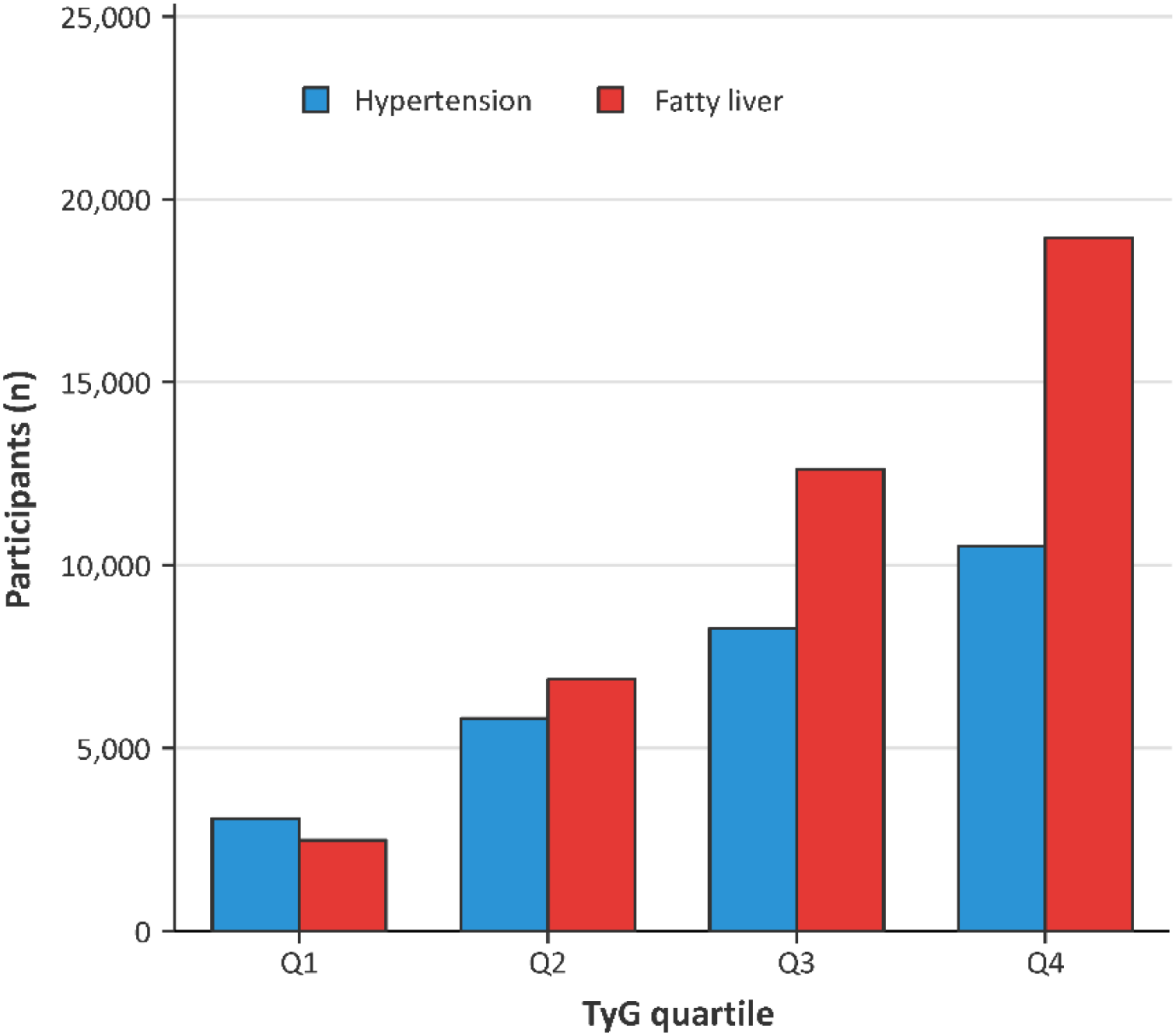
Distribution of hypertension and fatty liver across quartiles of the triglyceride–glucose index.

**Figure 2** shows the distribution of participants with hypertension and fatty liver across quartiles of the triglyceride–glucose index. The numbers of participants with hypertension and fatty liver both increased progressively from the lowest quartile (Q1) to the highest quartile (Q4), with the highest counts observed in Q4 and the lowest in Q1.

### Association between the TyG index and hypertension

**Table 2** presents the association between the TyG index and hypertension based on logistic regression models. Each 1-unit increase in the TyG index was significantly associated with higher odds of hypertension in the non-adjusted model (OR = 2.73, 95% CI: 2.66–2.79). The association remained significant after adjustment for age and sex (Model 1: OR = 2.06, 95% CI: 2.00–2.12) and after further adjustment for marital status, ethnicity, physical activity, alcohol consumption, and smoking status (Model 2: OR = 2.09, 95% CI: 2.03–2.15). When analyzed by TyG quartiles, compared with participants in the lowest quartile (Q1), those in Q2, Q3, and Q4 had significantly higher odds of hypertension, with adjusted ORs of 1.49 (95% CI: 1.41–1.57), 2.05 (95% CI: 1.94–2.16), and 3.06 (95% CI: 2.91–3.23), respectively (P < 0.001).

**Table 2:** Association between the TyG index and hypertension.

Exposure	Non-adjusted model OR (95% CI)	Model 1 OR (95% CI)	Model 2 OR (95% CI)	P value
TyG index (per unit increase)	2.73 (2.66–2.79)	2.06 (2.00–2.12)	2.09 (2.03–2.15)	<0.001
TyG quartiles				<0.001
Q1 (lowest)	Reference	Reference	Reference	
Q2	2.13 (2.03–2.23)	1.48 (1.41–1.56)	1.49 (1.41–1.57)	
Q3	3.45 (3.30–3.61)	2.05 (1.95–2.15)	2.05 (1.94–2.16)	
Q4 (highest)	5.26 (5.03–5.50)	3.01 (2.87–3.16)	3.06 (2.91–3.23)	

Odds ratios (ORs) and 95% confidence intervals (CIs) were estimated using logistic regression models. Missing data in physical activity, smoking status, and alcohol consumption were handled using the missing-indicator method.

Non-adjusted model: no covariates adjusted.

Model 1: adjusted for sex and age.

Model 2: further adjusted for marital status, ethnicity, physical activity, alcohol consumption, and smoking status.

### Association between the TyG index and fatty liver

**Table 3** shows a strong positive association between the TyG index and fatty liver. Each 1-unit increase in the TyG index was associated with higher odds of fatty liver in the non-adjusted model (OR = 8.52, 95% CI: 8.28–8.78), Model 1 (OR = 7.16, 95% CI: 6.94–7.38), and Model 2 (OR = 7.05, 95% CI: 6.82–7.28). Similar results were observed across TyG quartiles. Compared with Q1, the adjusted ORs for fatty liver were 2.91 (95% CI: 2.76–3.07) for Q2, 6.76 (95% CI: 6.42–7.12) for Q3, and 17.83 (95% CI: 16.90–18.82) for Q4 (P < 0.001).

**Table 3:** Association between the TyG index and fatty liver.

Exposure	Non-adjusted model OR (95% CI)	Model 1 OR (95% CI)	Model 2 OR (95% CI)	P value
TyG index (per unit increase)	8.52 (8.28–8.78)	7.16 (6.94–7.38)	7.05 (6.82–7.28)	<0.001
TyG quartiles				<0.001
Q1 (lowest)	Reference	Reference	Reference	
Q2	3.35 (3.19–3.52)	2.94 (2.79–3.09)	2.91 (2.76–3.07)	
Q3	8.56 (8.16–8.98)	6.90 (6.57–7.24)	6.76 (6.42–7.12)	
Q4 (highest)	24.31 (23.15–25.52)	18.31 (17.41–19.25)	17.83 (16.90–18.82)	

Odds ratios (ORs) and 95% confidence intervals (CIs) were estimated using logistic regression models. Missing data in physical activity, smoking status, and alcohol consumption were handled using the missing-indicator method.

Non-adjusted model: no covariates adjusted.

Model 1: adjusted for sex and age.

Model 2: further adjusted for marital status, ethnicity, physical activity, alcohol consumption, and smoking status.

### Mediation analysis

Mediation analysis suggested that fatty liver might statistically account for part of the observed association between the TyG index and hypertension (**Table 4**). The total effect was 0.087 (95% CI: 0.084–0.091; P < 0.001), the indirect effect was 0.021 (95% CI: 0.019–0.023; P < 0.001), and the direct effect was 0.067 (95% CI: 0.063–0.071; P < 0.001). The proportion statistically accounted for by fatty liver was 23.50% (95% CI: 21.39%–25.96%; P < 0.001).

**Table 4:** Mediation analysis of the association between the TyG index and hypertension through fatty liver.

Effect	Estimate	95% CI	P value
Total effect	0.087	0.084–0.091	<0.001
Indirect effect	0.021	0.019–0.023	<0.001
Direct effect	0.067	0.063–0.071	<0.001
Proportion statistically accounted for, %	23.50	21.39–25.96	<0.001

The mediation analysis was performed using generalized linear models with a probit link for the outcome model. For the binary outcome, the total, indirect, and direct effect estimates represent differences in the predicted probability of hypertension under the treatment contrast specified in the mediation analysis. Estimates were obtained using nonparametric bootstrap with 1,000 resamples and percentile-based 95% confidence intervals. The indirect and direct effects shown are average effects from the mediation analysis output. The models were adjusted for sex, age, marital status, ethnicity, physical activity, alcohol consumption, and smoking status. Because of the cross-sectional design, these results should be interpreted as a statistical decomposition of the observed association rather than evidence of a causal mediating effect.

### Sensitivity analyses

Additional sensitivity analyses with further adjustment for family history of hypertension and self-reported dietary preference variables yielded results that were generally consistent with the primary analyses, with the association between the TyG index and hypertension, the association between the TyG index and fatty liver, and the mediation results of fatty liver remaining materially unchanged (**Supplementary Tables S1**-**S3**).

## Discussion

In this large cross-sectional health examination study, we found that a higher TyG index was significantly associated with hypertension. Participants in the highest TyG quartile had substantially higher odds of hypertension than those in the lowest quartile, indicating a clear dose–response relationship. The TyG index was also strongly associated with fatty liver. In addition, mediation analysis suggested that fatty liver might account for part of the observed association between the TyG index and hypertension, with 23.50% of the association statistically accounted for by fatty liver.

Our findings are consistent with previous studies reporting associations between the TyG index and hypertension or fatty liver. Ruiz-García et al. (18) reported that a higher TyG index was independently associated with hypertension in individuals with metabolic syndrome, supporting the link between insulin resistance and elevated blood pressure. Gao et al. (19) further showed that the TyG-BMI index predicted hypertension among patients with fatty liver disease, highlighting the interplay between metabolic dysfunction and blood pressure regulation. Similarly, Fan et al. (20) observed a significant association between the TyG index and hepatic steatosis, although the magnitude of association differed across studies. Such differences may be related to variations in TyG categorization or study populations. In addition, a meta-analysis by Nayak et al. (21) confirmed the TyG index as a reliable marker of metabolic disorders, further supporting the relevance of our findings. Importantly, our study adds to the existing literature by exploring the possible contribution of fatty liver to the association between the TyG index and hypertension.

Several biological mechanisms may help explain the observed associations. The TyG index is widely recognized as a surrogate marker of insulin resistance and may also reflect broader disturbances in glucose and lipid metabolism (22). Insulin resistance may contribute to renal sodium retention, sympathetic nervous system activation, and endothelial dysfunction, all of which are associated with increased vascular resistance and elevated blood pressure (23). In addition, insulin resistance is closely linked to chronic low-grade inflammation and oxidative stress, which may further impair vascular function and promote vascular remodeling (24).

Fatty liver is closely related to insulin resistance, systemic inflammation, and dysregulated lipid metabolism, all of which are key features of cardiometabolic dysfunction (25, 26). In addition, fatty liver has been associated with increased production of pro-inflammatory cytokines, altered adipokine secretion, and abnormalities in hepatic insulin signaling. These alterations may contribute to broader systemic metabolic and inflammatory disturbances, impair endothelial function and vascular homeostasis, and thereby relate to higher blood pressure levels (27, 28).

However, the proportion statistically accounted for by fatty liver was modest, suggesting that fatty liver may represent only one component of the observed association between the TyG index and hypertension (29, 30). Other mechanisms, particularly systemic inflammation, visceral adiposity, and arterial stiffness, may also contribute to this association (31–34). Given the cross-sectional design, these findings should be interpreted as statistical associations rather than evidence of causality.

This study has several strengths. First, it included a large population undergoing routine health examinations, which provided substantial statistical power. Second, we adjusted for a prespecified set of demographic and lifestyle covariates and further conducted sensitivity analyses with additional adjustment for family history of hypertension and self-reported dietary preference, allowing a more robust evaluation of the observed associations. Third, to our knowledge, this study is among the few to explore the possible contribution of fatty liver to the association between the TyG index and hypertension, thereby extending the current understanding of the relationship between dysregulated glucose and lipid metabolism, insulin resistance, and hypertension.

However, several limitations should also be considered. First, because of the cross-sectional design, the temporal sequence among the TyG index, fatty liver, and hypertension could not be determined, and no causal inference can be made. Accordingly, the mediation analysis should be interpreted as a statistical exploration of the observed association rather than evidence of a causal mediating effect. Second, although multiple covariates were adjusted for and additional sensitivity analyses further included family history of hypertension and self-reported dietary preference variables, residual confounding from unmeasured or incompletely measured factors, such as detailed dietary intake, socioeconomic status, sleep duration, mental health, medication adherence, and genetic predisposition, cannot be completely excluded. Third, physical activity, smoking status, and alcohol consumption were based on self-reported questionnaire data, which may have introduced measurement error. In particular, physical activity was assessed using a relatively simple categorical measure with limited resolution, which may have led to misclassification and reduced our ability to fully account for its potential confounding effect. Fourth, fatty liver was identified based on ultrasound imaging during routine health examinations rather than more sensitive or specific methods such as MRI or histological confirmation, which may have led to some degree of misclassification. In addition, ultrasound could not distinguish between different etiologies of fatty liver, including non-alcoholic and alcoholic fatty liver disease. Such misclassification may also have affected the estimated statistical mediation effect. Finally, this was a single-center study based on a health examination population, and most participants were of Han ethnicity. Such a study population may overrepresent relatively healthier individuals or those with better access to preventive health services, and may not fully reflect the characteristics of community-based or high-risk populations. Therefore, the findings may not be fully generalizable to populations from other geographic regions, ethnic backgrounds, or clinical settings.

## Conclusion

In this health examination population, a higher TyG index was significantly associated with hypertension, and fatty liver may account for part of the observed association. Given the cross-sectional design, these findings should be interpreted cautiously, and further longitudinal studies are needed to clarify this relationship.

## Data Availability

The raw data supporting the conclusions of this article will be made available by the authors, without undue reservation.
