# Supplementary material for: Association between the triglyceride–glucose index and hypertension: potential role of fatty liver in a cross-sectional study
Source: Front Endocrinol (Lausanne). 2026 May 8;17:1826006. doi: 10.3389/fendo.2026.1826006 (PMC13193974; doi:10.3389/fendo.2026.1826006)
Supplement: Supplementary file 1 [file DataSheet1.docx]

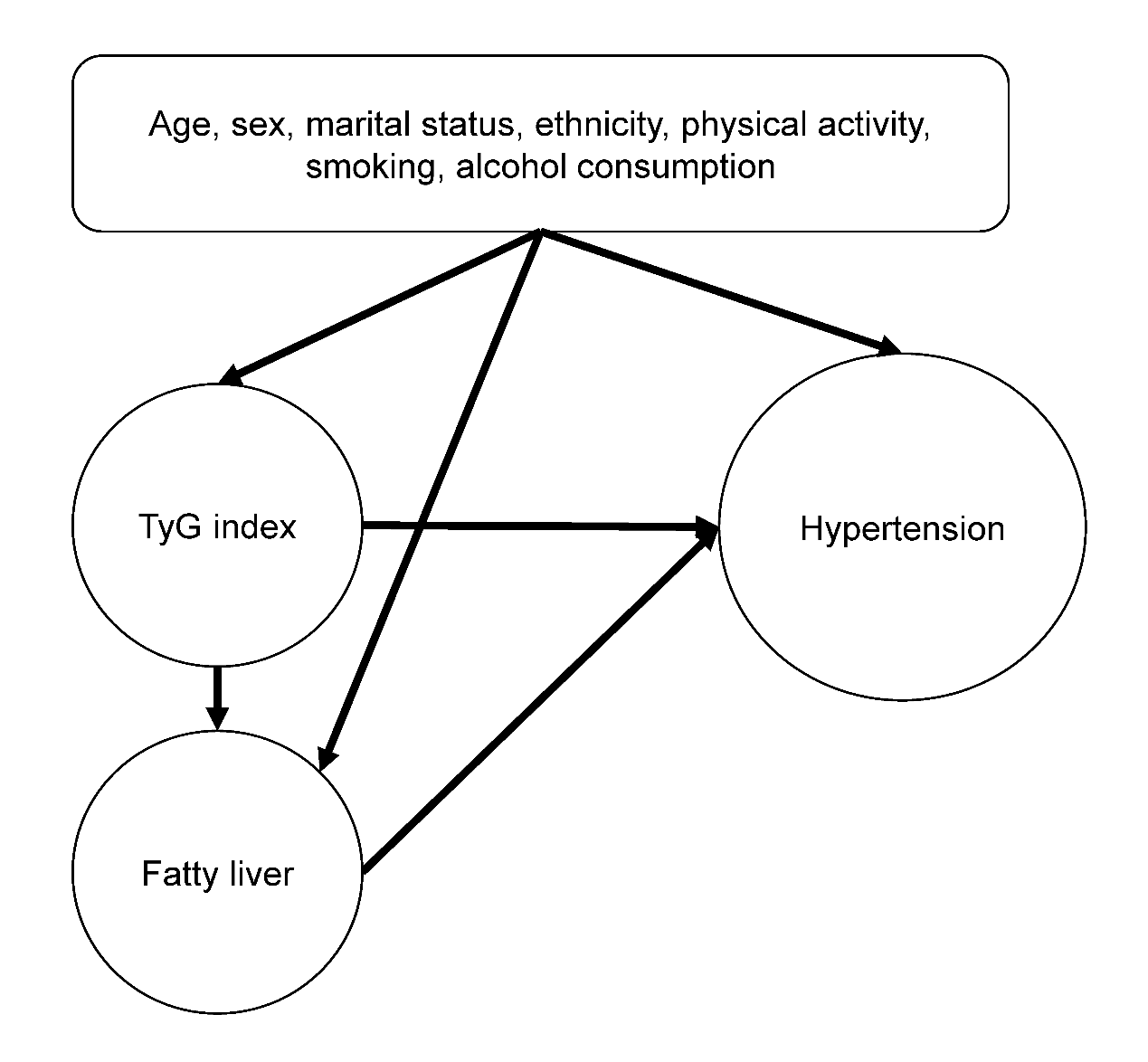


Figure S1. Directed acyclic graph (DAG) illustrating the hypothesized relationships among the TyG index, fatty liver, hypertension, and covariates. Age, sex, marital status, ethnicity, physical activity, smoking, and alcohol consumption were considered potential confounders and were included in the primary adjustment set. Fatty liver was examined as a proposed statistical mediator of the association between the TyG index and hypertension. Given the cross-sectional design, this DAG reflects hypothesized relationships based on prior knowledge rather than confirmed temporal ordering.
