# Supplementary material for: Association between the triglyceride–glucose index and hypertension: potential role of fatty liver in a cross-sectional study
Source: Front Endocrinol (Lausanne). 2026 May 8;17:1826006. doi: 10.3389/fendo.2026.1826006 (PMC13193974; doi:10.3389/fendo.2026.1826006)
Supplement: Supplementary file 2 [file DataSheet2.docx]

**Supplementary material**

Supplementary Tables S1–S3. Sensitivity analyses further adjusted for family history of hypertension and self-reported dietary preference variables

**Table S1.** Association between the TyG index and hypertension

| Exposure | Non-adjusted model OR (95% CI) | Model 1 OR (95% CI) | Model 2 OR (95% CI) | P value |
| --- | --- | --- | --- | --- |
| TyG index (per unit increase) | 2.73 (2.66–2.79) | 2.06 (2.00–2.12) | 2.08 (2.02–2.15) | <0.001 |
| TyG quartiles |  |  |  | <0.001 |
| Q1 (lowest) | Reference | Reference | Reference |  |
| Q2 | 2.13 (2.03–2.23) | 1.48 (1.41–1.56) | 1.49 (1.41–1.57) |  |
| Q3 | 3.45 (3.30–3.61) | 2.05 (1.95–2.15) | 2.05 (1.94–2.16) |  |
| Q4 (highest) | 5.26 (5.03–5.50) | 3.01 (2.87–3.16) | 3.06 (2.90–3.22) |  |

Odds ratios (ORs) and 95% confidence intervals (CIs) were estimated using logistic regression models. Missing data in physical activity, smoking status, alcohol consumption, family history of hypertension, and dietary preference were handled using the missing-indicator method.
Non-adjusted model: no covariates adjusted.
Model 1: adjusted for sex and age.
Model 2: further adjusted for marital status, ethnicity, physical activity, alcohol consumption, smoking status, family history of hypertension, and dietary preference.

**Table S2.** Association between the TyG index and fatty liver

| Exposure | Non-adjusted model OR (95% CI) | Model 1 OR (95% CI) | Model 2 OR (95% CI) | P value |
| --- | --- | --- | --- | --- |
| TyG index (per unit increase) | 8.52 (8.28–8.78) | 7.16 (6.94–7.38) | 7.08 (6.85–7.31) | <0.001 |
| TyG quartiles |  |  |  | <0.001 |
| Q1 (lowest) | Reference | Reference | Reference |  |
| Q2 | 3.35 (3.19–3.52) | 2.94 (2.79–3.09) | 2.92 (2.77–3.08) |  |
| Q3 | 8.56 (8.16–8.98) | 6.90 (6.57–7.24) | 6.78 (6.43–7.14) |  |
| Q4 (highest) | 24.31 (23.15–25.52) | 18.31 (17.41–19.25) | 17.91 (16.97–18.91) |  |

Odds ratios (ORs) and 95% confidence intervals (CIs) were estimated using logistic regression models. Missing data in physical activity, smoking status, alcohol consumption, family history of hypertension, and dietary preference were handled using the missing-indicator method.
Non-adjusted model: no covariates adjusted.
Model 1: adjusted for sex and age.
Model 2: further adjusted for marital status, ethnicity, physical activity, alcohol consumption, smoking status, family history of hypertension, and dietary preference.

**Table S3.** Mediation analysis of the association between the TyG index and hypertension through fatty liver

| Effect | Estimate | 95% CI | P value |
| --- | --- | --- | --- |
| Total effect | 0.087 | 0.084–0.091 | <0.001 |
| Indirect effect | 0.021 | 0.019–0.023 | <0.001 |
| Direct effect | 0.066 | 0.063–0.070 | <0.001 |
| Proportion statistically accounted for, % | 23.68 | 21.56–26.18 | <0.001 |

The mediation analysis was performed using generalized linear models with a probit link for the outcome model. For the binary outcome, the total, indirect, and direct effect estimates represent differences in the predicted probability of hypertension under the treatment contrast specified in the mediation analysis. Estimates were obtained using nonparametric bootstrap with 1,000 resamples and percentile-based 95% confidence intervals. The models were adjusted for sex, age, marital status, ethnicity, physical activity, alcohol consumption, smoking status, family history of hypertension, and self-reported dietary preference. Missing data in physical activity, smoking status, alcohol consumption, family history of hypertension, and self-reported dietary preference were handled using the missing-indicator method. Because of the cross-sectional design, the mediation results should be interpreted as a statistical exploration of the observed association.
